# Advances in tumor immunomodulation based on nanodrug delivery systems

**DOI:** 10.3389/fimmu.2023.1297493

**Published:** 2023-12-01

**Authors:** Bo Wang, Yue Zhang, Xunzhe Yin

**Affiliations:** ^1^ Department of Integrated Chinese and Western Medicine, Jilin Cancer Hospital, Changchun, China; ^2^ State Key Laboratory of Electroanalytical Chemistry, Changchun Institute of Applied Chemistry, Chinese Academy of Sciences, Changchun, China

**Keywords:** nanodrug delivery systems, tumor immunotherapy, tumor immunomodulation, tumor microenvironment, immune antitumor effect

## Abstract

Immunotherapy is a therapeutic approach that employs immunological principles and techniques to enhance and amplify the body’s immune response, thereby eradicating tumor cells. Immunotherapy has demonstrated effective antitumor effects on a variety of malignant tumors. However, when applied to humans, many immunotherapy drugs fail to target lesions with precision, leading to an array of adverse immune-related reactions that profoundly limit the clinical application of immunotherapy. Nanodrug delivery systems enable the precise delivery of immunotherapeutic drugs to targeted tissues or specific immune cells, enhancing the immune antitumor effect while reducing the number of adverse reactions. A nanodrug delivery system provides a feasible strategy for activating the antitumor immune response by the following mechanisms: 1) increased targeting and uptake of vaccines by DCs, which enhances the efficacy of the immune response; 2) increased tumor cell immunogenicity; 3) regulation of TAMs and other cells by, for example, regulating the polarization of TAMs and interfering with TAN formation, and ECM remodeling by CAFs; and 4) interference with tumor immune escape signaling pathways, namely, the PD-1/PD-L1, FGL1/LAG-3 and IDO signaling pathways. This paper reviews the progress of nanodrug delivery system research with respect to tumor immunotherapy based on tumor immunomodulation over the last few years, discussing the promising future of these delivery systems under this domain.

## 1 Introduction

Cancer is a grave health concern that poses a significant threat to human life. Currently, the diagnosis and treatment of cancer are arduous challenges (1). According to the latest epidemiological data, in 2020, the number of new cancer cases worldwide approximated to 19.3 million, with an associated death toll of 10 million. According to estimations, the global cancer burden is projected to rise to 28.4 million cases by 2040, representing a 47% increase compared to the burden in 2020 (2). At present, the principal approaches to cancer therapy encompass surgical intervention, radiotherapy, chemotherapy, targeted therapy, and immunotherapy. As a new tumor treatment method, immunotherapy is based on immunological principles leveraged to activate and promote the enhancement of the body’s immune system, thus facilitating an immune response to effectively eradicate tumor cells (3). Compared with conventional treatment, such as chemotherapy, immunotherapy shows a superior curative effect, a prolonged duration of action, and a lower incidence of adverse effects. Immunotherapy has revolutionized the treatment of multiple cancers (4), and it is the focus of recent cancer treatment research (5).

The effectiveness of clinical immunotherapy is hindered by the intricate mechanisms of tumor immune escape, resulting in a low positive response rate (6). Immunotherapy shows a profound therapeutic effect on only a portion of patients (7). Moreover, the distribution of immunotherapy drugs always encompasses various tissues and organs of the body, suggesting a lack of precise tumor targeting that may result in immune-related adverse reactions throughout the body (8, 9). Finding effective immunotherapy regulation methods, increasing the positive effect of immunotherapy, and reducing the incidence of immune-related adverse reactions are hot spots in current immunotherapy research (10).

Nanodrug delivery systems refer to nanocarriers that carry therapeutic drugs for *in vivo* delivery, and they have the advantages of preventing burst release and off-target effects of drugs, exhibiting desirable pharmacokinetic characteristics and flexibly controlling drug release (11). Combining cancer therapeutic drugs with nanocarriers can enable drugs to reach a target site or specific immune cells more accurately, leading to more effective immune responses, higher drug efficacy, and reduced incidence rates of adverse reactions (12). In recent years, many studies on the immunotherapeutic regulation of nanodrug delivery systems have been reported, and great therapeutic effects have been achieved (13). However, due to obstacles involving relevant basic research, production conditions, cost control and clinical trials, the pharmacokinetic study of some nanodrug delivery systems is insufficient, resulting in the current low conversion rate. We believe that the problems of achieving the long-term stability, effectiveness and safety of nanodrug delivery systems themselves, as well as the types of nanodrug delivery systems materials, challenges of industrialization, and cost of preparation methods should be solved one by one. Only in this way can we help break through this bottleneck. This paper reviews the successful application of nanodrug delivery systems within the realm of cancer research immunotherapy regulation over the past several years, the related treatment strategies, and the main challenges to this field and potential development directions.

## 2 Tumor immunotherapy

Tumor immunotherapy seeks to achieve the objective of stimulating or mobilizing the body’s immune system to generate an immune response that is capable of eradicating tumor cells (14). According to a mechanism classification system, tumor immunotherapy regimens can be categorized as “passive immunotherapy” and “active immunotherapy”. Passive immunotherapy refers to treatment that directly kills tumors mediated by anti-immune checkpoint-blocking antibodies and cytokines or immune cells expressing immune checkpoints. Passive immunotherapy mainly includes cytokine therapy, immune checkpoint inhibitors and adoptive cell therapy (15). Active immunotherapy refers to therapy that specifically activates the human autoimmune system and induces an active antitumor immune response. Tumor vaccines are the main types of therapies representing active immunotherapy (16).

**Figure 1:**
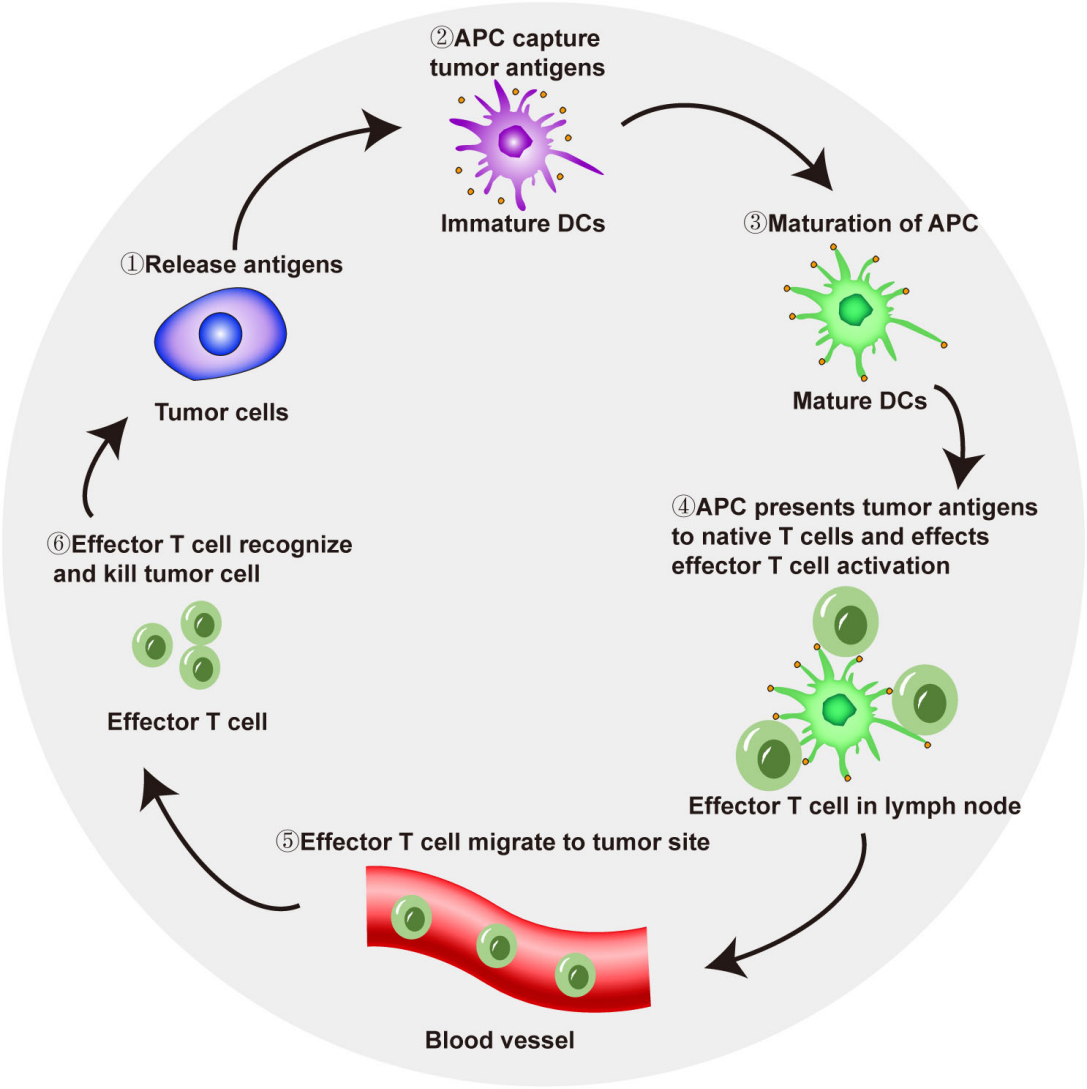
Mechanism of T cell-mediated antitumor immune response activation.

In tumor immunotherapy, T-cell-mediated immune response activation follows several key steps. Tumor cells release antigens that are taken up by antigen-presenting cells (APCs). APCs reach local lymph nodes through the lymphatic system and then present antigens to naive T cells through the major histocompatibility complex I (MHC I) pathway, thereby triggering the initiation and activation of effector T cells. Effector T cells reach a local tumor site through the blood flow and recognize and kill tumor cells (17, 18). Dead tumor cells release tumor-specific antigens, and APCs capture and take up these antigens, which also activate effector T cells, completing the cancer-immunity cycle (**Figure 1**). The active participation of CD4+ T cells is also needed for immune response activation. CD4+ T cells are important in the human immune system. They can bind to the non-polypeptide region of MHC II molecules and participate in the signal transduction of antigen recognition by T cell antigen receptors. In tumor immune responses, CD4+ T cells can activate CD8+ T cells through a variety of mechanisms, so that CD8+ T cells can differentiate into cytotoxic T lymphocytes while maintaining and strengthening the antitumor response. On the other hand, even in the absence of CD8+ T cells, CD4+ T cells can also kill tumor cells directly by mechanisms involving IFN-γ (19).

However, due to factors such as defective tumor antigen release, impaired T-cell priming in local lymph nodes, and tumor immunosuppressive signals (such as the downregulation of MHC expression by tumor cells) that enable tumor cells to escape immune surveillance, the cancer-immunity cycle may be impaired, causing tumor progression (20). Therefore, because of dysfunction in the cancer-immunity cycle, formulating personalized immunotherapy leads to better therapeutic effects for patients (21).

## 3 Overview of nanodrug delivery systems for cancer treatment

In recent years, with the wide application of nanotechnology in medicine, nanodrug delivery systems have been rapidly developed (22). Consequently, these systems can be classified as either naturally occurring natural carriers and synthetic carriers, based on their respective origins. These systems can be categorized into three subtypes: organic nanocarriers, inorganic nanocarriers and composite nanocarriers based on their composition (23). Organic nanocarriers consist of organic materials and include lipid-based carriers, viral capsids, polysaccharides and protein particles, while inorganic nanocarriers include metal nanoparticles such as gold or silver nanoparticles, ceramic nanoparticles, quantum dots, and carbon nanoparticles (24). As novel nanocarriers continue to emerge, the clinical and translational applications of nanomedicine will be expanded (25). To date, nanodrug delivery systems have shown many advantages. First, a nanodrug delivery system can reduce the number or degree of adverse reactions induced by chemotherapy drugs. Chemotherapy drugs usually have disadvantages such as low water solubility, instability under physiological conditions and can induce drug resistance and high toxicity. When chemotherapeutic drugs are integrated into nanodrug delivery systems by covalent bonding, physical packing, electrostatic forces or coordination complexation, the limitations of these chemotherapeutic drugs can be reduced (26, 27). The commonly used nanocarriers mainly include proteins, nucleic acids, small-molecule chemotherapeutics, and imaging agents (28–30). Second, the size effect of the nanodrug delivery system also affects the pharmacokinetics, cellular uptake rate, and the penetration and accumulation of drugs in tumor tissues (31, 32). Third, a nanodrug delivery system can also simultaneously deliver tracer drugs, enabling the integration of tumor disease diagnosis and treatment (33). Finally, a nanodrug delivery system may carry multiple therapeutic drugs, thereby achieving the superposition effects of the therapeutic drugs (34).

Nanodrug delivery systems are usually divided into active and passive targeting effects, both of which have their own advantages and disadvantages (35–37). The active targeting effect refers to a combination of nanoparticles with overexpressed tumor cell receptors to achieve the targeting effect. The commonly used targeting receptors include mainly folate, transferrin and epidermal growth factor receptor (38). The passive targeting effect refers to the accumulation of nanoparticles that move through the circulatory systems of the human body and then accumulate in tumors, thereby playing a therapeutic role. Therefore, the passive targeting effect is particularly dependent on the physiological characteristics of the tumor microenvironment (39). A research showed that nanoparticles with diameters in the range of 40-400 nm stayed in the circulatory system for a long time, enabling their high accumulation in tumors, and reduced renal clearance (40). Moreover, the passive targeting effect may result in a random targeting effect, which may lead to insufficient diffusion of drugs in tumors.

**Figure 2:**
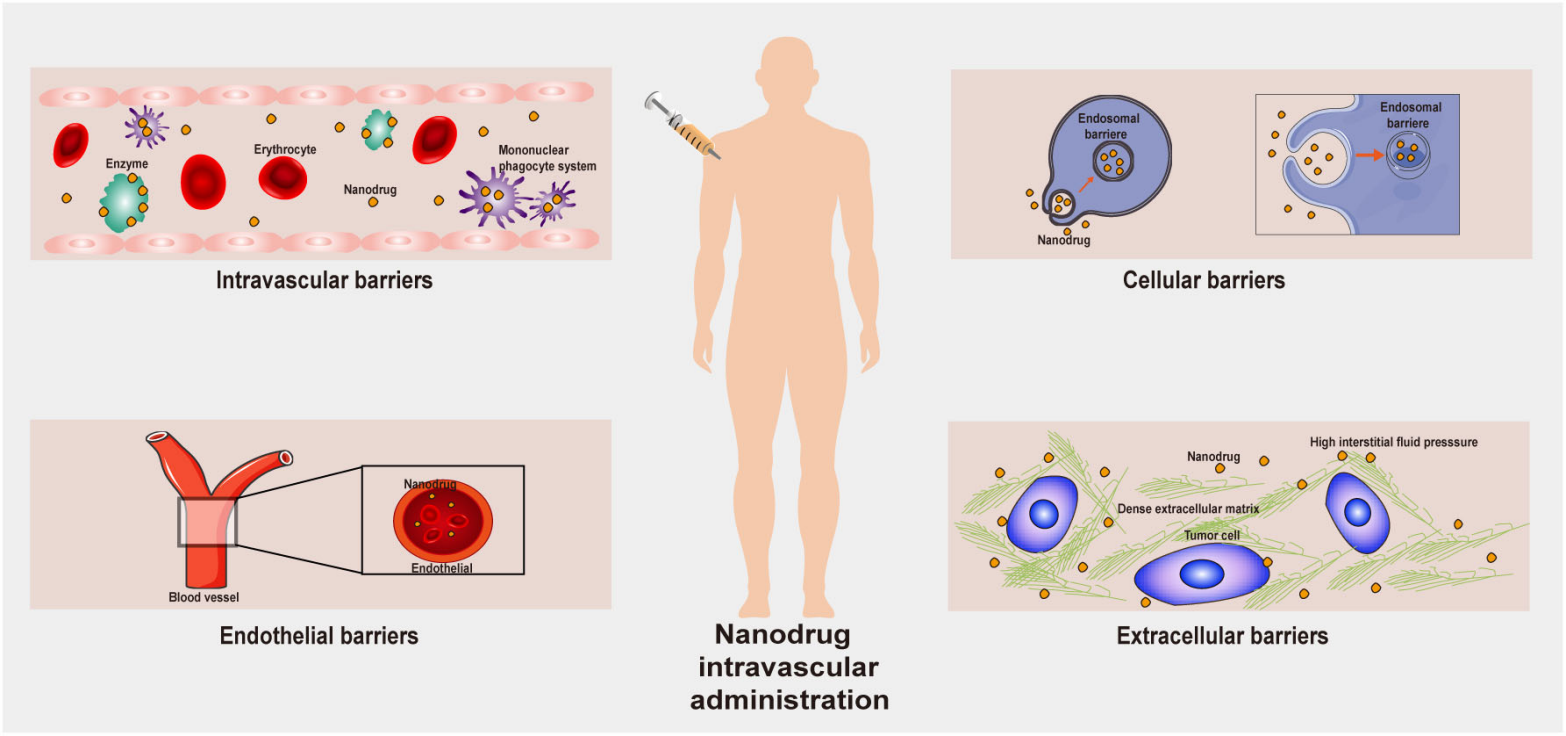
Biological barriers that a nanodrug may encounter in the human body.

After a nanodrug delivery system enters the human body, it needs to overcome many obstacles to achieve a therapeutic effect on tumor cells (41, 42). First, nanoparticles present in the blood circulatory system interact in a remarkable fashion with the plasma proteins, eventually being sequestered by the reticuloendothelial system (RES). Therefore, circulating nanoparticles must first escape the RES before they can accumulate in tumor tissues. When a nanodrug reaches a tumor tissue, although the tumor blood vessels allow the nanodrug to accumulate, parietal cells may limit nanodrug passage through openings in the capillary wall, thereby decreasing the rate of convective transport of the nanodrug. Moreover, the dense extracellular matrix (ECM) can inhibit the passive diffusion of nanodrugs due to high osmotic pressure. All these factors present obstacles to the transportation of nanodrugs through tumor blood vessels (**Figure 2**). The transportation of nanodrug delivery systems is a thorny problem. It remains difficult to correctly specify nanodrug delivery systems, and further follow-up research is still needed to accurately adjust the size, shape and hydrophilicity/hydrophobicity of the nanodrug delivery systems to solve the transportation problem.

## 4 Tumor immunotherapy regulation via a nanodrug delivery system

### 4.1 Nanodrug delivery systems increase tumor cell vaccine uptake rates and efficiency

Tumor vaccines can specifically activate the human autoimmune system and contribute to the regulation of the antitumor immune response (43). Tumor vaccines are mainly divided into cell vector vaccines, protein vaccines, peptide vaccines and nucleic acid vaccines (44). Nanodrug delivery systems encapsulate tumor antigens and adjuvants into the same carrier (called nano vaccines, particle vaccines, or nanoparticle vaccines) and deliver them into the same APC, preventing immune tolerance caused by the absence of an adjuvant (45). In addition, a nanodrug delivery system also significantly increases the uptake efficiency of tumor antigens by APCs, thereby increasing the antitumor immune response effect. To achieve a stronger immune response effect, nanoparticle vaccines need to meet several conditions, including effective antigen-loading capacity, efficient lymphatic drainage efficiency, and enhanced APC uptake capacity (46).

Dendritic cells (DCs) are important target cells of nanoparticle vaccines. They are important APCs that can take up, process and present antigens, produce cytokines and chemokines, and initiate T-cell-mediated immune responses (47). Specific ligands modified on the surface of nanoparticle vaccines target receptors on the surface of DCs, which increases the uptake efficiency of the DCs through specific endocytic pathways (48). J Chen developed mannose-modified PLL-RT (Man-PLL-RT)-mediated nanovaccines with DC-targeting ability. Man-PLL-RT with antigens (ovalbumin, OVA) and adjuvants (unmethylated cytosine-phosphate-guanine, CpG) coencapsulated by electrostatic interaction that facilitated antigen endocytosis, maturation and cross presentation by DCs (49).

Conventional tumor vaccines activate specific T cells through the action of DCs to induce immune responses indirectly. The efficacy of a tumor vaccine usually contingents upon the suboptimal activation of T cells. S Go developed a nanovaccine that enhanced the T-cell response through its interactions with DCs and T cells to treat cancer. This nanovaccine consisted of a cancer cell membrane nanoparticle (CCM-MPLA) decorated with monophosphoryl lipid A (MPLA). Researchers conjugated anti-CD28 antibodies (aCD28) to the CCM-MPLA particles to produce CCM-MPLA-aCD28 nanoparticles, which induced direct interactions between nanovaccines and tumor-specific T cells. Regardless of the presence or absence of DCs, this nanovaccine activated tumor-specific CD8 T cells, exhibiting more effective induction of tumor-specific CD8 T-cell responses and exhibiting high antitumor efficacy in tumor-bearing mice (50).

Activation of the stimulator of interference genes (STING) pathway enhanced the antigen-presenting efficiency of DCs, thereby promoting the antitumor immune response of T cells (51, 52). X Jiang developed a PC7A nanovaccine that activated STING. This vaccine induced antigen-specific T cells to undergo robust tumor infiltration, generate a strong antitumor T-cell response, and induce antitumor immune responses and showed higher efficacy when intratumorally administered compared to when it was subcutaneously injected (53).

In addition to utilizing receptor-mediated endocytosis to increase the uptake efficiency of nanovaccines by DCs, strategies based on macropinocytosis were used to increase the internalization efficiency of DCs (54). Macropinocytosis is a transient, actin-driven endocytic process based on membrane folding to create a vacuole that engulfs exogenous fluids and particles on a large scale into cells (55). In contrast to other cells, DCs show efficient uptake of exogenous antigens through macropinocytosis, which can potentially compensate for the insufficient of expression of specific receptors on DCs and thus increase their antigen uptake efficiency. C Yang developed a nanoparticulate vaccine based on a reactive oxygen species (ROS)-responsive nanoparticle core and macropinocytosis-inducing peptide-fused cancer membrane shell. The vaccine was taken up by DCs at a significantly higher rate via CXC-chemokine receptor type 4 (CXCR4)-mediated macropinocytosis and jointly promoted DCs maturation and the T-cell immune response by activating the STING pathway (56).

Spherical nucleic acids (SNAs) are capable of rapid uptake by antigen-presenting cells through receptor-mediated endocytosis. For innate immune responses, the unique adjuvant nucleic acid three-dimensional structure of the SNA shell can provide improved recognition of Toll-like receptors. Nanovaccines have been used in clinical practice for the treatment of prostate cancer, but the efficacy is high, and further research is imminent (57). SNA vaccines developed by researchers can improve the production and secretion of cytokines and can increase polyfunctional cytotoxic T cells and effector memory. In this nanovaccine, human prostate-specific membrane antigen or T-cell receptor γ alternate reading frame protein was integrated into the optimized structure, resulting in a high immune activation rate and a high cytolytic capacity of humanized mouse and human peripheral blood mononuclear cells (hPBMCs) (58). Some researchers have developed immunostimulatory SNA (IS-SNA) nanostructures composed of CpG oligonucleotides as an adjuvant and prostate cancer peptide antigen. This nanovaccine increases the codelivery of CpG and antigen to DCs, thereby improving the cross-priming of antitumor CD8 T cells and generating more effective antitumor immune responses (59).

### 4.2 Nanodrug delivery systems enhance tumor cell immunogenicity

**Figure 3:**
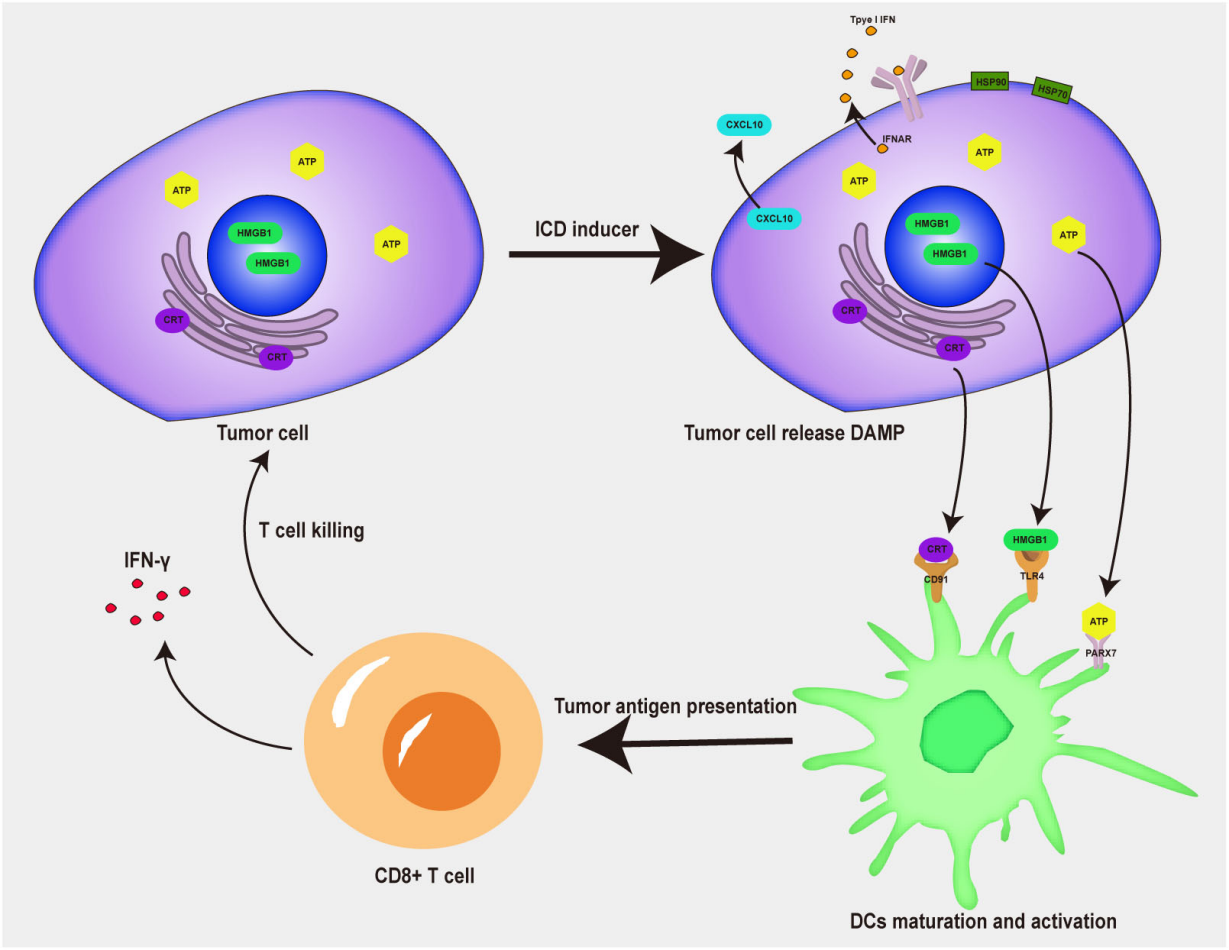
Process of ICD inducers inducing immune response.

Low immunogenicity and an immunosuppressive tumor microenvironment are major impediments to efficacious tumor immunotherapy (60). At present, some immunogenic cell death (ICD) inducers, have been confirmed to promote tumor immunotherapy by triggering ICD (61). When tumor cells undergo ICD, they release a variety of tumor-associated antigens and damage-associated molecular patterns (DAMPs) (62–64), such as high mobility group protein 1 (HMGB1), calreticulin (CRT), adenosine-triphosphate (ATP) and heat shock protein (HSP90α) (**Figure 3**). DAMP promotes the activation of APCs, induces the activation of antigen-specific T cells, promotes the intratumoral infiltration of immune cells, and thus enhances the immune response in tumors (65, 66). Compared with other therapies, DAMPs released by ICD inducers can mobilize immune stimulation, promote the maturation of DCs, and activate T cells, thus achieving a positive tumor chemoimmunotherapy effect (67). According to their ability to activate cell death or release DAMPs, ICD inducers can be divided into two different categories: type I and type II (68). Type I ICD inducers do not induce tumor cell death by increasing endoplasmic reticulum stress, but the effects of lateral endoplasmic reticulum stress produce immunogenicity; type II ICD inducers selectively target the endoplasmic reticulum and release danger and apoptosis signaling molecules because of ROS-dependent endoplasmic reticulum stress. Most chemotherapeutic drugs are type I ICD inducers, while radiotherapy and photodynamic therapy are type II ICD inducers. Due to strong adverse reactions and low tumor-targeting efficiency, ICD inducers use has been limited. A combination of a nanodrug delivery system and ICD inducers makes possible the clinical use of ICD inducers during tumor immunotherapy (69). S Liu developed a doxorubicin (DOX) and 4-(hydroxymethyl) phenylboronic acid pinacol ester (PBAP) prodrug polymer and encapsulated it with chlorin e6 (Ce6) in nanoparticles to obtain hyaluronidase (HAase) and HO dual-sensitive responsive nanoparticles (Ce6/HDP NPs). The NPs displayed efficient intratumoral accumulation and cellular internalization properties due to the active targeting of hyaluronic acid (HA). The strong ICD stimuli, which were induced by ROS production and GSH depletion, led to amplified immunogenicity to activate tumor immunotherapy. The DNA damage caused by the dual effects of chemotherapy and ROS production directly caused tumor cell apoptosis (70).

Radiotherapy, photodynamic therapy and sonodynamic therapy induce the ICD effect of tumor cells through the effects of ROS-induced endoplasmic reticulum stress (71). However, the hypoxic environment of tumors profoundly affects ROS production, which indirectly leads to an insufficient immune response to ICD. Alleviating hypoxia is a key problem that urgently needs to be solved to enable the use of nanodrug delivery systems (72). To solve this problem, M Wang created an albumin-based nanoplatform codelivering IR780, a NLG919 dimer and the hypoxia-activated prodrug tirapazamine (TPZ) as a dual enhancer of synergistic cancer therapy. TPZ-mediated chemotherapy by increasing the photodynamic therapy-induced tumor ICD rate, which induced a stronger antitumor immune response, including an increase in the number of tumor-specific cytotoxic T lymphocytes (73).

In addition to a ROS-based strategy, an endoplasmic reticulum targeting strategy can enhance the ICD effects (74, 75). H Luo found that inhibiting the endoplasmic reticulum-associated protein degradation pathway stimulated the ICD-induced lysate emission from dying esophageal cancer cells in a dose-dependent manner. Dual therapy with an endoplasmic reticulum-associated protein degradation inhibitor combined with medium-dose radiotherapy triggered an antitumor immune response by increasing the maturation and phagocytosis rates of DCs (76).

### 4.3 Nanodrug delivery systems regulate the tumor microenvironment

**Figure 4:**
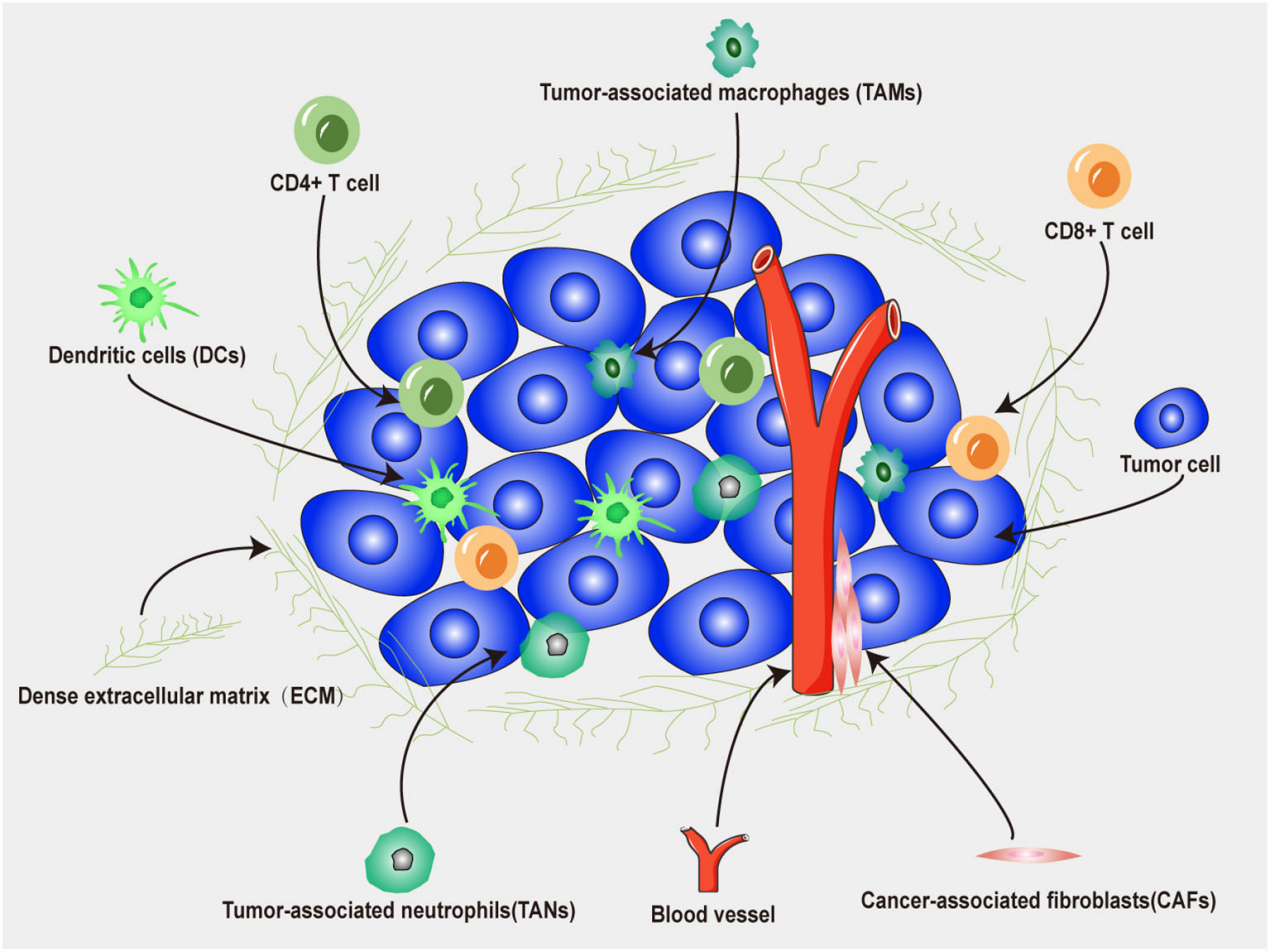
Main components of tumor microenvironment.

The tumor microenvironment (TME) plays a crucial role in the interaction between tumors and immunotherapy (77). As shown in **Figure 4**, the TME is the surrounding microenvironment in which tumor cells exist and is composed of neutrophils, DCs, T cells, fibroblasts, macrophages, microvessels, various signaling molecules and molecular cytokines (78). Tumor cells can release various cell signaling molecules, which in turn affect their immune microenvironment, induce immune tolerance, and inhibit antitumor immune responses (79). In addition, regulatory T (Treg) cells, tumor-associated macrophages, tumor-associated fibroblasts, and myeloid-derived suppressor cells in the TME can increase immunosuppressive effect and tumor cell evasion rate, further increasing the complexity of the TME (80). Therefore, a nanodrug delivery system can be designed to target immunosuppressive cells or pathways to reduce the immunosuppressive effect of the TME, which is anticipated to increase the efficacy of tumor immunotherapy (81).

#### 4.3.1 Regulation of tumor-associated macrophages

Tumor-associated macrophages (TAMs) are essential interstitial cells in the TME and are composed of two types of cells that either inhibit or promote tumor cell proliferation (82, 83). M1 macrophages have antitumor effects and secrete classical inflammatory cytokines to kill tumors by promoting tumor cell necrosis and immune cell infiltration into the TME (84). M2 macrophages mainly play a role in promoting tumor growth, invasion and metastasis by degrading the tumor ECM, destroying the basement membrane, promoting angiogenesis and recruiting immunosuppressive cells (85). The application of a nanodrug delivery system to regulate TAM activity can enhance the antitumor immune response (86). Commonly used therapeutic strategies include blocking macrophage recruitment, interfering with TAM survival programs, and remodeling M2-type TAMs into the M1 type TAMs (87). S Ha fabricated PLGA nanoparticles encapsulating baicalin and the melanoma antigen Hgp peptide fragment consisting of amino acids 25-33 by using the ultrasonic double-emulsion technique. The nanoparticles were loaded with CpG fragments, and M2pep and α-pep peptides were conjugated onto their surfaces to yield novel nanocomplexes. The nanocomplexes were effectively internalized by M2-phenotype TAMs *in vitro* and *in vivo*. The acidic lysosomal environment was observed to trigger the disintegration of the polydopamine on the nanoparticle surface, leading to the release of the payloads. The release of CpG from the tumor microenvironment is a critical factor in the transformation of M2-phenotype TAMs into M1-phenotype TAMs, leading to increased secretion of inflammatory cytokines. The decreased secretion of cytokines by TAMs subsequently suppresses tumor angiogenesis, enabling the tumor microenvironment to undergo significant changes (88).

Tumor cells secrete a variety of stimulatory factors, such as macrophage colony-stimulating factor, which can bind to the tyrosine kinase CSF receptor 1 on macrophages, leading to their conversion into the M2-phenotype macrophages. Therefore, blocking the CSF-1R signaling pathway can remodel the M2-phenotype to M1-phenotype macrophages (89). Y-W Chang generated a bifunctional protein by fusing interleukin-10 to ana anti-colony-stimulating factor-1 receptor-blocking antibody. The fusion protein demonstrated significant antitumor activity in multiple cancer models, especially models of head and neck cancer. This bifunctional protein not only led to the anticipated reduction in the number of TAMs but also triggered the proliferation, activation, and metabolic reprogramming of CD8 T cells (90).

#### 4.3.2 Interference with tumor-associated neutrophils

Neutrophils are important components of the immune system and essential immune cells that fight against microbial infection. Neutrophils account for a large proportion of immune cells infiltrating tumor tissues, which are called tumor-associated neutrophils (TANs) (91). TANs exert dual effects on tumors; namely, they show antitumor (the N1 subpopulation) and tumor-promoting activity (the N2 subpopulation). N1 subpopulation TANs induce antitumor activity through antibody-dependent cytotoxicity and other mechanisms, while N2 subpopulation TANs promote tumor growth by enhancing tumor cell metastasis, promoting angiogenesis and inhibiting the action of adaptive immune cells (92, 93).

The use of nanodrug delivery carriers to interfere with neutrophil development and function, destroy their immunosuppressive function and restore their anticancer properties has gradually become a promising therapeutic strategy. Using a nanodrug delivery system to interfere with the development and function of TANs, particularly their immunosuppressive function, and thus restore their anticancer effects is a promising therapeutic strategy (94, 95). Y Wang developed a nanovaccine constructed with SiPCCl-hybridized mesoporous silica with Fe(III)-captopril complexes and coated with the exfoliated membrane of mature DCs via H22-specific neoantigen stimulation. The nanovaccines actively target H22 tumors and induce ICD. Moreover, acid-triggered captopril release into the tumor microenvironment polarized protumoral N2 phenotype neutrophils into antitumor N1 phenotype neutrophils to increase the immune effects (96).

Enhancing the sensitivity of TANs and achieving more precise drug delivery contribute to more profound immune effects. H Dong proposed a novel concept that utilizes a nanoimmunotraining strategy to rapidly activate neutrophil tumor tropism and consequently enhance the targeting capacity of antitumor drugs. An evaluation of this strategy demonstrated significantly increased tumor-targeted accumulation of neutrophils harvested from nanoimmunotrained mice after either intraperitoneal or intravenous injection of a vaccine-like nano-CpG adjuvant, which led to the precise delivery of nanodrugs (97).

#### 4.3.3 Targeting tumor-associated fibroblasts

Cancer-associated fibroblasts (CAFs) in the tumor microenvironment can promote tumors by interacting with cancer cells (98). Moreover, CAFs can form a solid physical barrier by secreting ECM and other components, hindering the penetration and diffusion of nanodrugs in tumor tissues and reducing the infiltration rate of tumor-infiltrating lymphocytes (99). In addition, CAFs can induce an microenvironment immune tolerance by secreting cytokines (100).

Regulating the formation of CAFs, eliminating CAFs, remodeling CAFs and other strategies can increase the efficiency of nanodrug delivery and relieve TME immunosuppression to enhance antitumor immunotherapy (101, 102). Y Chen developed a nanoparticle that significantly inhibited tumor growth and metastasis by remodeling CAFs in the TME. Y Chen found that salvianolic acid B-loaded PEGylated liposomes (PEG-SAB-Lip) interfere with the activation of CAFs by inhibiting the secretion of TGF-β1. After inhibiting the activation of CAFs, the collagen deposition rate in tumors was reduced, and the penetration rate of nanoparticles in tumors was increased. These outcomes led to the high expression of cytokines and chemokines (CXCL9 and CXCL10) in T helper 1 (Th1) cells and the recruitment of CD4, CD8 T cells, and M1 macrophages to the tumor area (103).

## 5 Interference with the tumor immune escape signaling pathway

There are many immunosuppressive signaling pathways in TAMs. Malignant cells themselves, or lymphocytes that infiltrate tumors, can abnormally express a variety of immune checkpoint molecules, including PD-1/PD-L1, LAG-3, TIM3, and TIGIT, which inhibit the activation of antigen-specific T cells and facilitate tumor cell immune escape (104, 105). Immune regulatory molecules released by tumor cells, such as TGF-β and indoleamine 2,3-dioxygenase (IDO), can prevent effector T-cell function and even lead to T-cell exhaustion in tumors (106). Exhausted T cells become dysfunctional, are unable to produce cytotoxic effects, and lose the ability to produce antitumor cytokines such as IL-2 and TNF-α, which leads to a reduction in tumor immunotherapy efficiency (107, 108). Therefore, blocking the tumor cell immune escape signaling pathway with a nanodrug delivery system may activate or increase the antitumor immune response.

The upregulation of immune checkpoint molecules in TAM is a significant mechanism contributing to tumor immune evasion, and blocking this signaling pathway can enhance the endogenous antitumor immune effect of the body. Many studies have shown that using immune checkpoint inhibitors to block the PD-1/PD-L1 signaling pathway reversed the immunosuppression of the TME, restored T-cell antitumor activity, and enhanced tumor immunotherapy (109, 110). S Liu prepared atovaquone-loaded human serum albumin (HSA) nanoparticles stabilized via intramolecular disulfide bonds, calling them HSA-ATO NPs. These nanoparticles show excellent bioavailability, tumor targeting ability, and high biosafety. HSA-ATO NPs can promote intratumoral CD8 T-cell recruitment by alleviating hypoxia in the TME, thereby enhancing the efficacy of anti-PD-1 immunotherapy (111).

However, blocking only a single immune escape signaling pathway is not enough to induce a strong antitumor immune effect. FGL1/LAG-3 is a newly discovered immune escape signaling pathway. Similar to the PD-1/PD-L1 signaling pathway, it exerts a variety of biological regulatory effects on T cells (112). Dual blockade of the FGL1/LAG-3 and PD-1/PD-L1 signaling pathways greatly improved the T-cell killing ability of tumor cells. W-J Wan designed a new type of ROS-sensitive nanoparticle and loaded it with FGL1 short interfering RNA (siRNA; siFGL1) and PD-L1 siRNA (siPD-L1), which they formed from a stimulus-responsive polymer with poly-l-lysine-thioketal and modified cis-aconitate to facilitate nanoparticle endosomal escape. Furthermore, the administration of the tumor-penetrating peptide iRGD and ROS-responsive nanoparticles concurrently enhanced the delivery efficiency of siFGL1 and siPD-L1, leading to a significant reduction in the protein levels of FGL1 and PD-L1 in tumor cells (113).

IDO is a ferrous heme-containing oxidoreductase that can degrade tryptophan to yield kynurenine, which can directly inhibit the function of cytotoxic T lymphocytes increase Treg activity to play an immunosuppressive role (114). The tryptophan metabolism signaling pathway activated by IDO is important in promoting tumor cell immune escape. Inhibition of IDO prevented the inhibition of T-cell proliferation in the TME and activated or enhanced autoimmune function (115). C Yang developed a polycaprolactone-based nanoparticle to encapsulate the tryptanthrin derivative CY-1-4. These nanoparticles both induced ICD and inhibited IDO effects while regulating the formation of lymphocyte subsets in the spleen and tumor (116).

## 6 Conclusions and prospects

Immunotherapy has been a revolutionary treatment for cancer patients. However, due to the complex tumor cell immune escape mechanism, many problems to be solved to increase the efficacy of tumor immunotherapy; these problems include profound differences among individual patients, low rates of positive effects and adverse reactions. Nanodrug delivery systems can improve the pharmacokinetic characteristics of drugs, increase their bioavailability, and reduce their adverse reactions, suggesting their use in a novel approach to cancer immunotherapy. According to the mechanism underlying an activated antitumor immune response, a nanodrug delivery system provides a feasible strategy if it has the following effects: 1) increases the targeting and uptake of vaccines by DCs, thereby enhancing the efficacy of the immune response; 2) increases tumor cell immunogenicity; 3) regulates TAMs and other cells by, for example, regulating the polarization of TAMs and interfering with TAN formation and ECM remodeling by CAFs; and 4) interferes with tumor immune escape signaling pathways, namely, the PD-1/PD-L1, FGL1/LAG-3 and IDO signaling pathways. Although some achievements have been made in research on nanodrug delivery systems, their practical application still faces some urgent problems. 1) First, the main problems come from the lack of long-term stability, effectiveness and safety of nanodrug delivery systems. Other problems involve the types of delivery systems materials available, the industrialization of preparation methods, packaging and cost issues. The solutions to all of these problems will necessitate comprehensive and in-depth research in the corresponding disciplines or even between multiple disciplines. 2) Secondly, nanodrug delivery systems and their degradation products may not be pharmacologically inert substances, so there are inevitably some potential safety hazards. Current efforts to limit the toxicity of nanodrug delivery systems involve material modification, composition optimization and the development of new materials. 3) Thirdly, the pharmacokinetic behavior of nanodrug delivery systems does not fully satisfy clinical requirements, which also limits the clinical translation of nanodrug delivery systems. Therefore, the key to the application and development of nanodrug delivery systems is to design efficient, safe and intelligent nanodrug delivery systems and study their pharmacokinetics *in vivo* in detail. 4) Finally, tumor tissue has a highly heterogeneous microenvironment, and differences occur among different patients or tumors of the same patient at different times. Widespread tumor heterogeneity presents great difficulties for immunotherapy with nanodrug delivery systems. With the extensive study of genomics and tumor pathological mechanism, researchers should further study and screen for tumor-related markers and should develop more personalized nanodrug delivery systems from the molecular level to achieve better therapeutic effects. With the development of nanotechnology, nanomaterials will also be constantly updated and iterated. We believe that with the ongoing deepening of research and development of science and technology, the advantages of nanodrug delivery systems will be used more extensively in the clinical treatment of malignant tumor diseases and will become a powerful tool for humans to overcome cancer.

## Author contributions

BW: Conceptualization, Writing – original draft. YZ: Conceptualization, Investigation, Supervision, Writing – review & editing. XY: Conceptualization, Investigation, Supervision, Writing – original draft, Writing – review & editing.

## References

[1] Siegel RL Miller KD Wagle NS Jemal A. Cancer statistics, 2023. CA Cancer J Clin (2023) 73(1):17–48. doi: 10.3322/caac.21763 36633525

[2] Sung H Ferlay J Siegel RL Laversanne M Soerjomataram I Jemal A. Global cancer statistics 2020: GLOBOCAN estimates of incidence and mortality worldwide for 36 cancers in 185 countries. CA Cancer J Clin (2021) 71(3):209–49. doi: 10.3322/caac.21660 33538338

[3] Dagher OK Schwab RD Brookens SK Posey AD. Advances in cancer immunotherapies. Cell (2023) 186(8):1814–1814.e1. doi: 10.1016/j.cell.2023.02.039 37059073

[4] Bagchi S Yuan R Engleman EG. Immune checkpoint inhibitors for the treatment of cancer: clinical impact and mechanisms of response and resistance. Annu Rev Pathol-mech (2021) 16:223–49. doi: 10.1146/annurev-pathol-042020-042741 33197221

[5] Vivekanandhan S Bahr D Kothari A Ashary MA Baksh M Gabriel E. Immunotherapies in rare cancers. Mol Cancer (2023) 22(1):23. doi: 10.1186/s12943-023-01720-2 36726126 PMC9890725

[6] Martínez-Jiménez F Priestley P Shale C Baber J Rozemuller E Cuppen E. Genetic immune escape landscape in primary and metastatic cancer. Nat Genet (2023) 55(5):820–31. doi: 10.1038/s41588-023-01367-1 PMC10181939 37165135

[7] Taefehshokr S Parhizkar A Hayati S Mousapour M Mahmoudpour A Eleid L. Cancer immunotherapy: Challenges and limitations. Pathol Res Pract (2022) 229:153723. doi: 10.1016/j.prp.2021.153723 34952426

[8] Wang SJ Dougan SK Dougan M. Immune mechanisms of toxicity from checkpoint inhibitors. Trends Cancer (2023) 9(7):543–53. doi: 10.1016/j.trecan.2023.04.002 PMC10330206 37117135

[9] Kennedy LB Salama A. A review of cancer immunotherapy toxicity. CA Cancer J Clin (2020) 70(2):86–104. doi: 10.3322/caac.21596 31944278

[10] Wahida A Buschhorn L Fröhling S Jost PJ Schneeweiss A Lichter P. The coming decade in precision oncology: six riddles. Nat Rev Cancer (2023) 23(1):43–54. doi: 10.1038/s41568-022-00529-3 36434139

[11] Cheng Z Li M Dey R Chen Y. Nanomaterials for cancer therapy: current progress and perspectives. J Hematol Oncol (2021) 14(1):85. doi: 10.1186/s13045-021-01096-0 34059100 PMC8165984

[12] Li Q Shi Z Zhang F Zeng W Zhu D Mei L. Symphony of nanomaterials and immunotherapy based on the cancer-immunity cycle. Acta Pharm Sin B (2022) 12(1):107–34. doi: 10.1016/j.apsb.2021.05.031 PMC8799879 35127375

[13] Yang B Gao J Pei Q Xu H Yu H. Engineering prodrug nanomedicine for cancer immunotherapy. Adv Sci (Weinh) (2020) 7(23):2002365. doi: 10.1002/advs.202002365 33304763 PMC7709995

[14] Hiam-Galvez KJ Allen BM Spitzer MH. Systemic immunity in cancer. Nat Rev Cancer (2021) 21(6):345–59. doi: 10.1038/s41568-021-00347-z PMC8034277 33837297

[15] Schlake T Thess A Thran M Jordan I. mRNA as novel technology for passive immunotherapy. Cell Mol Life Sci (2019) 76(2):301–28. doi: 10.1007/s00018-018-2935-4 PMC6339677 30334070

[16] Saxena M van der Burg SH Melief C Bhardwaj N. Therapeutic cancer vaccines. Nat Rev Cancer (2021) 21(6):360–78. doi: 10.1038/s41568-021-00346-0 33907315

[17] Ager A. Cancer immunotherapy: T cells and neutrophils working together to attack cancers. Cell (2023) 186(7):1304–6. doi: 10.1016/j.cell.2023.03.005 37001496

[18] Chen C Liu X Chang CY Wang HY Wang RF. The interplay between T cells and cancer: the basis of immunotherapy. Genes (Basel) (2023) 14(5):1008. doi: 10.3390/genes14051008 37239368 PMC10218245

[19] Basu A Ramamoorthi G Albert G Gallen C Beyer A Snyder C. Differentiation and regulation of T_H_ cells: A balancing act for cancer immunotherapy. Front Immunol (2021) 12:669474. doi: 10.3389/fimmu.2021.669474 34012451 PMC8126720

[20] Lei X Lei Y Li JK Du WX Li RG Yang J. Immune cells within the tumor microenvironment: Biological functions and roles in cancer immunotherapy. Cancer Lett (2020) 470:126–33. doi: 10.1016/j.canlet.2019.11.009 31730903

[21] Majzner RG Mackall CL. Tumor antigen escape from CAR T-cell therapy. Cancer Discovery (2018) 8(10):1219–26. doi: 10.1158/2159-8290.CD-18-0442 30135176

[22] Nasir A Khan A Li J Naeem M Khalil A Khan K. Nanotechnology, A tool for diagnostics and treatment of cancer. Curr Top Med Chem (2021) 21(15):1360–76. doi: 10.2174/1568026621666210701144124 34218784

[23] Harshita Wu HF Kailasa SK. Recent advances in nanomaterials-based optical sensors for detection of various biomarkers (inorganic species, organic and biomolecules). Luminescence (2023) 38(7):954–98. doi: 10.1002/bio.4353 35929140

[24] Tan YY Yap PK Xin Lim GL Mehta M Chan Y Ng SW. Perspectives and advancements in the design of nanomaterials for targeted cancer theranostics. Chem Biol Interact (2020) 329:109221. doi: 10.1016/j.cbi.2020.109221 32768398

[25] Yu M Yang W Yue W Chen Y. Targeted cancer immunotherapy: nanoformulation engineering and clinical translation. Adv Sci (Weinh) (2022) 9(35):e2204335. doi: 10.1002/advs.202204335 36257824 PMC9762307

[26] Huang P Wang X Liang X Yang J Zhang C Kong D. Nano-, micro-, and macroscale drug delivery systems for cancer immunotherapy. Acta Biomater (2019) 85:1–26. doi: 10.1016/j.actbio.2018.12.028 30579043

[27] Wei G Wang Y Yang G Wang Y Ju R. Recent progress in nanomedicine for enhanced cancer chemotherapy. Theranostics (2021) 11(13):6370–92. doi: 10.7150/thno.57828 PMC8120226 33995663

[28] Torres-Vanegas JD Cruz JC Reyes LH. Delivery systems for nucleic acids and proteins: barriers, cell capture pathways and nanocarriers. Pharmaceutics (2021) 13(3):428. doi: 10.3390/pharmaceutics13030428 33809969 PMC8004853

[29] Wang QY Xu YS Zhang NX Dong ZP Zhao BN Liu LC. Phenylboronic ester-modified anionic micelles for ROS-stimuli response in HeLa cell. Drug Delivery (2020) 27(1):681–90. doi: 10.1080/10717544.2020.1748761 PMC7269054 32393138

[30] Ehlerding EB Grodzinski P Cai W Liu CH. Big potential from small agents: nanoparticles for imaging-based companion diagnostics. ACS Nano (2018) 12(3):2106–21. doi: 10.1021/acsnano.7b07252 PMC5878691 29462554

[31] Ravindran S Suthar JK Rokade R Deshpande P Singh P Pratinidhi A. Pharmacokinetics, metabolism, distribution and permeability of nanomedicine. Curr Drug Metab (2018) 19(4):327–34. doi: 10.2174/1389200219666180305154119 29512450

[32] Li X Montague EC Pollinzi A Lofts A Hoare T. Design of smart size-, surface-, and shape-switching nanoparticles to improve therapeutic efficacy. Small (2022) 18(6):e2104632. doi: 10.1002/smll.202104632 34936204

[33] Jin W Dong C Yang D Zhang R Jiang T Wu D. Nano-carriers of combination tumor physical stimuli-responsive therapies. Curr Drug Delivery (2020) 17(7):577–87. doi: 10.2174/1567201817666200525004225 32448102

[34] Xiang J Liu K Xu H Zhao Z Piao Y Shao S. Dual synergistic tumor-specific polymeric nanoparticles for efficient chemo-immunotherapy. Adv Sci (Weinh) (2023) 10(29):e2301216. doi: 10.1002/advs.202301216 37551065 PMC10582463

[35] Xu M Han X Xiong H Gao Y Xu B Zhu G. Cancer nanomedicine: emerging strategies and therapeutic potentials. Molecules (2023) 28(13):5145. doi: 10.3390/molecules28135145 37446806 PMC10343932

[36] Li R Zheng K Yuan C Chen Z Huang M. Be active or not: the relative contribution of active and passive tumor targeting of nanomaterials. Nanotheranostics (2017) 1(4):346–57. doi: 10.7150/ntno.19380 PMC5646738 29071198

[37] Attia MF Anton N Wallyn J Omran Z Vandamme TF. An overview of active and passive targeting strategies to improve the nanocarriers efficiency to tumour sites. J Pharm Pharmacol (2019) 71(8):1185–98. doi: 10.1111/jphp.13098 31049986

[38] Srinivasarao M Low PS. Ligand-targeted drug delivery. Chem Rev (2017) 117(19):12133–64. doi: 10.1021/acs.chemrev.7b00013 28898067

[39] Clemons TD Singh R Sorolla A Chaudhari N Hubbard A Iyer KS. Distinction between active and passive targeting of nanoparticles dictate their overall therapeutic efficacy. Langmuir (2018) 34(50):15343–9. doi: 10.1021/acs.langmuir.8b02946 30441895

[40] Shi Y van der Meel R Chen X Lammers T. The EPR effect and beyond: Strategies to improve tumor targeting and cancer nanomedicine treatment efficacy. Theranostics (2020) 10(17):7921–4. doi: 10.7150/thno.49577 PMC7359085 32685029

[41] de Lázaro I Mooney DJ. Obstacles and opportunities in a forward vision for cancer nanomedicine. Nat Mater (2021) 20(11):1469–79. doi: 10.1038/s41563-021-01047-7 34226688

[42] Sindhwani S Syed AM Ngai J Kingston BR Maiorino L Rothschild J. The entry of nanoparticles into solid tumours. Nat Mater (2020) 19(5):566–75. doi: 10.1038/s41563-019-0566-2 31932672

[43] Morse MA Gwin WR Mitchell DA. Vaccine therapies for cancer: then and now. Target Oncol (2021) 16(2):121–52. doi: 10.1007/s11523-020-00788-w PMC7845582 33512679

[44] DeMaria PJ Bilusic M. Cancer vaccines. Hematol Oncol Clin North Am (2019) 33(2):199–214. doi: 10.1016/j.hoc.2018.12.001 30832995

[45] Bowen WS Svrivastava AK Batra L Barsoumian H Shirwan H. Current challenges for cancer vaccine adjuvant development. Expert Rev Vaccines (2018) 17(3):207–15. doi: 10.1080/14760584.2018.1434000 PMC6093214 29372660

[46] Schunke J Mailänder V Landfester K Fichter M. Delivery of immunostimulatory cargos in nanocarriers enhances anti-tumoral nanovaccine efficacy. Int J Mol Sci (2023) 24(15):12174. doi: 10.3390/ijms241512174 37569548 PMC10419017

[47] Wooster AL Girgis LH Brazeale H Anderson TS Wood LM Lowe DB. Dendritic cell vaccine therapy for colorectal cancer. Pharmacol Res (2021) 164:105374. doi: 10.1016/j.phrs.2020.105374 33348026 PMC7867624

[48] Fucikova J Hensler M Kasikova L Lanickova T Pasulka J Rakova J. An autologous dendritic cell vaccine promotes anticancer immunity in patients with ovarian cancer with low mutational burden and cold tumors. Clin Cancer Res (2022) 28(14):3053–65. doi: 10.1158/1078-0432.CCR-21-4413 35536547

[49] Chen J Fang H Hu Y Wu J Zhang S Feng Y. Combining mannose receptor mediated nanovaccines and gene regulated PD-L1 blockade for boosting cancer immunotherapy. Bioact Mater (2022) 7:167–80. doi: 10.1016/j.bioactmat.2021.05.036 PMC8379363 34466725

[50] Go S Jung M Lee S Moon S Hong J Kim C. A personalized cancer nanovaccine that enhances T-cell responses and efficacy through dual interactions with dendritic cells and T cells. Adv Mater (2023) 29:e2303979. doi: 10.1002/adma.202303979 37515819

[51] Zhang Y Shen T Zhou S Wang W Lin S Zhu G. pH-responsive STING-activating DNA nanovaccines for cancer immunotherapy. Adv Ther (Weinh) (2020) 3(9):2000083. doi: 10.1002/adtp.202000083 34337143 PMC8323737

[52] Zhou L Hou B Wang D Sun F Song R Shao Q. Engineering polymeric prodrug nanoplatform for vaccination immunotherapy of cancer. Nano Lett (2020) 20(6):4393–402. doi: 10.1021/acs.nanolett.0c01140 32459969

[53] Jiang X Wang J Zheng X Liu Z Zhang X Li Y. Intratumoral administration of STING-activating nanovaccine enhances T cell immunotherapy. J Immunother Cancer (2022) 10(5):e003960. doi: 10.1136/jitc-2021-003960 35623658 PMC9150169

[54] Qiu Z Liu W Zhu Q Ke K Zhu Q Jin W. The role and therapeutic potential of macropinocytosis in cancer. Front Pharmacol (2022) 13:919819. doi: 10.3389/fphar.2022.919819 36046825 PMC9421435

[55] Mylvaganam S Freeman SA Grinstein S. The cytoskeleton in phagocytosis and macropinocytosis. Curr Biol (2021) 31(10):R619–32. doi: 10.1016/j.cub.2021.01.036 34033794

[56] Yang C Zhang F Chen F Chang Z Zhao Y Shao D. Biomimetic nanovaccines potentiating dendritic cell internalization *via* CXCR4-mediated macropinocytosis. Adv Healthc Mater (2023) 12(5):e2202064. doi: 10.1002/adhm.202202064 36416257

[57] Liu S Yu CY Wei H. Spherical nucleic acids-based nanoplatforms for tumor precision medicine and immunotherapy. Mater Today Bio (2023) 22:100750. doi: 10.1016/j.mtbio.2023.100750 PMC10400933 37545568

[58] Teplensky MH Dittmar JW Qin L Wang S Evangelopoulos M Zhang B. Spherical nucleic acid vaccine structure markedly influences adaptive immune responses of clinically utilized prostate cancer targets. Adv Healthc Mater (2021) 10(22):e2101262. doi: 10.1002/adhm.202101262 34494382 PMC8599645

[59] Qin L Wang S Dominguez D Long A Chen S Fan J. Development of spherical nucleic acids for prostate cancer immunotherapy. Front Immunol (2020) 11:1333. doi: 10.3389/fimmu.2020.01333 32733447 PMC7362897

[60] Riley RS June CH Langer R Mitchell MJ. Delivery technologies for cancer immunotherapy. Nat Rev Drug Discovery (2019) 18(3):175–96. doi: 10.1038/s41573-018-0006-z PMC6410566 30622344

[61] Ghiringhelli F Rébé C. Using immunogenic cell death to improve anticancer efficacy of immune checkpoint inhibitors: from basic science to clinical application. Immunol Rev (2023) 18. doi: 10.1111/imr.13263 37593811

[62] DoltonGRiusCWallASzomolayBBianchiVGallowayS. Targeting of multiple tumor-associated antigens by individual T cell receptors during successful cancer immunotherapy. Cell (2023) 186(16):3333–3349.e27. doi: 10.1016/j.cell.2023.06.020 37490916

[63] Roh JS Sohn DH. Damage-associated molecular patterns in inflammatory diseases. Immune Netw (2018) 18(4):e27. doi: 10.4110/in.2018.18.e27 30181915 PMC6117512

[64] Ahmed A Tait S. Targeting immunogenic cell death in cancer. Mol Oncol (2020) 14(12):2994–3006. doi: 10.1002/1878-0261.12851 33179413 PMC7718954

[65] Lee SM Kim P You J Kim EH. Role of damage-associated molecular pattern/cell death pathways in vaccine-induced immunity. Viruses (2021) 13(12):2340. doi: 10.3390/v13122340 34960608 PMC8708515

[66] Fucikova J Kepp O Kasikova L Petroni G Yamazaki T Liu P. Detection of immunogenic cell death and its relevance for cancer therapy. Cell Death Dis (2020) 11(11):1013. doi: 10.1038/s41419-020-03221-2 33243969 PMC7691519

[67] Zhao Z Wang X Wang J Li Y Lin W Lu K. A nanobody-bioorthogonal catalyst conjugate triggers spatially confined prodrug activation for combinational chemo-immunotherapy. J Med Chem (2023) 66(17):11951–64. doi: 10.1021/acs.jmedchem.3c00557 37590921

[68] Huang J Duan F Xie C Xu J Zhang Y Wang Y. Microbes mediated immunogenic cell death in cancer immunotherapy. Immunol Rev (2023) 8. doi: 10.1111/imr.13261 37553793

[69] Duan X Chan C Lin W. Nanoparticle-mediated immunogenic cell death enables and potentiates cancer immunotherapy. Angew Chem Int Ed Engl (2019) 58(3):670–80. doi: 10.1002/anie.201804882 PMC7837455 30016571

[70] Liu S Kong X Fang Y He Z Wu H Ji J. A dual-sensitive nanoparticle-mediated deepening synergistic therapy strategy involving DNA damage and ICD stimuli to treat triple-negative breast cancer. Biomater Sci (2023) 11(18):6325–41. doi: 10.1039/d3bm00781b 37555273

[71] Zhou J Wang G Chen Y Wang H Hua Y Cai Z. Immunogenic cell death in cancer therapy: Present and emerging inducers. J Cell Mol Med (2019) 23(8):4854–65. doi: 10.1111/jcmm.14356 PMC6653385 31210425

[72] Yang B Chen Y Shi J. Reactive oxygen species (ROS)-based nanomedicine. Chem Rev (2019) 119(8):4881–985. doi: 10.1021/acs.chemrev.8b00626 30973011

[73] Wang M He M Zhang M Xue S Xu T Zhao Y. Controllable hypoxia-activated chemotherapy as a dual enhancer for synergistic cancer photodynamic immunotherapy. Biomaterials (2023) 301:122257. doi: 10.1016/j.biomaterials.2023.122257 37531778

[74] Shi Y Wang S Wu J Jin X You J. Pharmaceutical strategies for endoplasmic reticulum-targeting and their prospects of application. J Control Release (2021) 329:337–52. doi: 10.1016/j.jconrel.2020.11.054 33290795

[75] Wu H Chen W Chen Z Li X Wang M. Novel tumor therapy strategies targeting endoplasmic reticulum-mitochondria signal pathways. Ageing Res Rev (2023) 88:101951. doi: 10.1016/j.arr.2023.101951 37164161

[76] Luo H Sun Y Wang L Liu H Zhao R Song M. Targeting endoplasmic reticulum associated degradation pathway combined with radiotherapy enhances the immunogenicity of esophageal cancer cells. Cancer Biol Ther (2023) 24(1):2166763. doi: 10.1080/15384047.2023.2166763 36907982 PMC10026871

[77] Traba J Sack MN Waldmann TA Anton OM. Immunometabolism at the nexus of cancer therapeutic efficacy and resistance. Front Immunol (2021) 12:657293. doi: 10.3389/fimmu.2021.657293 34079545 PMC8166297

[78] de Visser KE Joyce JA. The evolving tumor microenvironment: From cancer initiation to metastatic outgrowth. Cancer Cell (2023) 41(3):374–403. doi: 10.1016/j.ccell.2023.02.016 36917948

[79] Sexauer D Gray E Zaenker P. Tumour- associated autoantibodies as prognostic cancer biomarkers- a review. Autoimmun Rev (2022) 21(4):103041. doi: 10.1016/j.autrev.2022.103041 35032685

[80] Zhang Y Zhang Z. The history and advances in cancer immunotherapy: understanding the characteristics of tumor-infiltrating immune cells and their therapeutic implications. Cell Mol Immunol (2020) 17(8):807–21. doi: 10.1038/s41423-020-0488-6 PMC7395159 32612154

[81] Cheng Y Song S Wu P Lyu B Qin M Sun Y. Tumor associated macrophages and TAMs-based anti-tumor nanomedicines. Adv Healthc Mater (2021) 10(18):e2100590. doi: 10.1002/adhm.202100590 34292673

[82] Chen S Saeed A Liu Q Jiang Q Xu H Xiao GG. Macrophages in immunoregulation and therapeutics. Signal Transduct Target Ther (2023) 8(1):207. doi: 10.1038/s41392-023-01452-1 37211559 PMC10200802

[83] Mishra AK Malonia SK. Advancing cellular immunotherapy with macrophages. Life Sci (2023) 328:121857. doi: 10.1016/j.lfs.2023.121857 37307965

[84] Yunna C Mengru H Lei W Weidong C. Macrophage M1/M2 polarization. Eur J Pharmacol (2020) 877:173090. doi: 10.1016/j.ejphar.2020.173090 32234529

[85] Kadomoto S Izumi K Mizokami A. Macrophage polarity and disease control. Int J Mol Sci (2021) 23(1):144. doi: 10.3390/ijms23010144 35008577 PMC8745226

[86] Kumari N Choi SH. Tumor-associated macrophages in cancer: recent advancements in cancer nanoimmunotherapies. J Exp Clin Cancer Res (2022) 41(1):68. doi: 10.1186/s13046-022-02272-x 35183252 PMC8857848

[87] Zhao C Pang X Yang Z Wang S Deng H Chen X. Nanomaterials targeting tumor associated macrophages for cancer immunotherapy. J Control Release (2022) 341:272–84. doi: 10.1016/j.jconrel.2021.11.028 34813877

[88] Han S Wang W Wang S Yang T Zhang G Wang D. Tumor microenvironment remodeling and tumor therapy based on M2-like tumor associated macrophage-targeting nano-complexes. Theranostics (2021) 11(6):2892–916. doi: 10.7150/thno.50928 PMC7806477 33456579

[89] Muñoz-Garcia J Cochonneau D Télétchéa S Moranton E Lanoe D Brion R. The twin cytokines interleukin-34 and CSF-1: masterful conductors of macrophage homeostasis. Theranostics (2021) 11(4):1568–93. doi: 10.7150/thno.50683 PMC7778581 33408768

[90] Chang YW Hsiao HW Chen JP Tzeng SF Tsai CH Wu CY. A CSF-1R-blocking antibody/IL-10 fusion protein increases anti-tumor immunity by effectuating tumor-resident CD8 T cells. Cell Rep Med (2023) 4(8):101154. doi: 10.1016/j.xcrm.2023.101154 37586318 PMC10439276

[91] Que H Fu Q Lan T Tian X Wei X. Tumor-associated neutrophils and neutrophil-targeted cancer therapies. Biochim Biophys Acta Rev Cancer (2022) 1877(5):188762. doi: 10.1016/j.bbcan.2022.188762 35853517

[92] Masucci MT Minopoli M Carriero MV. Tumor associated neutrophils. Their role in tumorigenesis, metastasis, prognosis and therapy. Front Oncol (2019) 9:1146. doi: 10.3389/fonc.2019.01146 31799175 PMC6874146

[93] Arvanitakis K Mitroulis I Germanidis G. Tumor-associated neutrophils in hepatocellular carcinoma pathogenesis, prognosis, and therapy. Cancers (Basel) (2021) 13(12):2899. doi: 10.3390/cancers13122899 34200529 PMC8228651

[94] Chellappan DK Yee LW Xuan KY Kunalan K Rou LC Jean LS. Targeting neutrophils using novel drug delivery systems in chronic respiratory diseases. Drug Dev Res (2020) 81(4):419–36. doi: 10.1002/ddr.21648 32048757

[95] Chu D Dong X Shi X Zhang C Wang Z. Neutrophil-based drug delivery systems. Adv Mater (2018) 30(22):e1706245. doi: 10.1002/adma.201706245 29577477 PMC6161715

[96] Wang Y Zhao Q Zhao B Zheng Y Zhuang Q Liao N. Remodeling tumor-associated neutrophils to enhance dendritic cell-based HCC neoantigen nano-vaccine efficiency. Adv Sci (Weinh) (2022) 9(11):e2105631. doi: 10.1002/advs.202105631 35142445 PMC9009112

[97] Dong H Li Y Liu Y Wen Y Zou Z Yang T. A nano-immunotraining strategy to enhance the tumor targeting of neutrophils via *in vivo* pathogen-mimicking stimulation. Biomater Sci (2019) 7(12):5238–46. doi: 10.1039/c9bm01278h 31602440

[98] Zhang M Fang Y Fu X Liu J Liu Y Zhu Z. Cancer-associated fibroblasts nurture LGR5 marked liver tumor-initiating cells and promote their tumor formation, growth, and metastasis. Cancer Med (2023) 12(17):18032–49. doi: 10.1002/cam4.6408 PMC10524013 37578396

[99] Ai W Liu T Lv C Feng X Wang Q. Modulation of cancer-associated fibroblasts by nanodelivery system to enhance efficacy of tumor therapy. Nanomed (Lond) (2023) 18(15):1025–39. doi: 10.2217/nnm-2023-0088 37584613

[100] Biffi G Tuveson DA. Diversity and biology of cancer-associated fibroblasts. Physiol Rev (2021) 101(1):147–76. doi: 10.1152/physrev.00048.2019 PMC7864232 32466724

[101] Chen Y McAndrews KM Kalluri R. Clinical and therapeutic relevance of cancer-associated fibroblasts. Nat Rev Clin Oncol (2021) 18(12):792–804. doi: 10.1038/s41571-021-00546-5 34489603 PMC8791784

[102] Chen X Song E. Turning foes to friends: targeting cancer-associated fibroblasts. Nat Rev Drug Discovery (2019) 18(2):99–115. doi: 10.1038/s41573-018-0004-1 30470818

[103] Chen Y Hu M Wang S Wang Q Lu H Wang F. Nano-delivery of salvianolic acid B induces the quiescence of tumor-associated fibroblasts via interfering with TGF-β1/Smad signaling to facilitate chemo- and immunotherapy in desmoplastic tumor. Int J Pharm (2022) 623:121953. doi: 10.1016/j.ijpharm.2022.121953 35753535

[104] Yi M Zheng X Niu M Zhu S Ge H Wu K. Combination strategies with PD-1/PD-L1 blockade: current advances and future directions. Mol Cancer (2022) 21(1):28. doi: 10.1186/s12943-021-01489-2 35062949 PMC8780712

[105] Anderson AC Joller N Kuchroo VK. Lag-3, tim-3, and TIGIT: co-inhibitory receptors with specialized functions in immune regulation. Immunity (2016) 44(5):989–1004. doi: 10.1016/j.immuni.2016.05.001 27192565 PMC4942846

[106] Prendergast GC Malachowski WJ Mondal A Scherle P Muller AJ. Indoleamine 2,3-dioxygenase and its therapeutic inhibition in cancer. Int Rev Cell Mol Biol (2018) 336:175–203. doi: 10.1016/bs.ircmb.2017.07.004 29413890 PMC6054468

[107] Liu Y Zhou N Zhou L Wang J Zhou Y Zhang T. IL-2 regulates tumor-reactive CD8 T cell exhaustion by activating the aryl hydrocarbon receptor. Nat Immunol (2021) 22(3):358–69. doi: 10.1038/s41590-020-00850-9 33432230

[108] De Ridder K Locy H Piccioni E Zuazo MI Awad RM Verhulst S. TNF-α-secreting lung tumor-infiltrated monocytes play a pivotal role during anti-PD-L1 immunotherapy. Front Immunol (2022) 13:811867. doi: 10.3389/fimmu.2022.811867 35493461 PMC9046849

[109] Ai L Xu A Xu J. Roles of PD-1/PD-L1 pathway: signaling, cancer, and beyond. Adv Exp Med Biol (2020) 1248:33–59. doi: 10.1007/978-981-15-3266-5_3 32185706

[110] Liu S Wang H Shao X Chen H Chao S Zhang Y. Advances in PD-1 signaling inhibition-based nano-delivery systems for tumor therapy. J Nanobiotechnol (2023) 21(1):207. doi: 10.1186/s12951-023-01966-4 PMC10318732 37403095

[111] Wang S Zhou X Zeng Z Sui M Chen L Feng C. Atovaquone-HSA nano-drugs enhance the efficacy of PD-1 blockade immunotherapy by alleviating hypoxic tumor microenvironment. J Nanobiotechnol (2021) 19(1):302. doi: 10.1186/s12951-021-01034-9 PMC8487475 34600560

[112] Wang J Sanmamed MF Datar I Su TT Ji L Sun J. Fibrinogen-like protein 1 is a major immune inhibitory ligand of LAG-3. Cell (2019) 176(1-2):334–47. doi: 10.1016/j.cell.2018.11.010 PMC6365968 30580966

[113] Wan WJ Huang G Wang Y Tang Y Li H Jia CH. Coadministration of iRGD peptide with ROS-sensitive nanoparticles co-delivering siFGL1 and siPD-L1 enhanced tumor immunotherapy. Acta Biomater (2021) 136:473–84. doi: 10.1016/j.actbio.2021.09.040 34571271

[114] Ogbechi J Huang YS Clanchy F Pantazi E Topping LM Darlington LG. Modulation of immune cell function, IDO expression and kynurenine production by the quorum sensor 2-heptyl-3-hydroxy-4-quinolone (PQS). Front Immunol (2022) 13:1001956. doi: 10.3389/fimmu.2022.1001956 36389710 PMC9650388

[115] Guo Y Liu Y Wu W Ling D Zhang Q Zhao P. Indoleamine 2,3-dioxygenase (Ido) inhibitors and their nanomedicines for cancer immunotherapy. Biomaterials (2021) 276:121018. doi: 10.1016/j.biomaterials.2021.121018 34284200

[116] Yang C He B Zheng Q Wang D Qin M Zhang H. Nano-encapsulated tryptanthrin derivative for combined anticancer therapy via inhibiting indoleamine 2,3-dioxygenase and inducing immunogenic cell death. Nanomed (Lond) (2019) 14(18):2423–40. doi: 10.2217/nnm-2019-0074 31549585

